# Advancements in high-resolution 3D microscopy analysis of endosomal morphology in postmortem Alzheimer’s disease brains

**DOI:** 10.3389/fnins.2023.1321680

**Published:** 2024-01-16

**Authors:** Shannon E. Rose, C. Andrew Williams, Dale W. Hailey, Swati Mishra, Amanda Kirkland, C. Dirk Keene, Gwenn A. Garden, Suman Jayadev, Jessica E. Young

**Affiliations:** ^1^Department of Laboratory Medicine and Pathology, University of Washington, Seattle, WA,United States; ^2^Institute for Stem Cell and Regenerative Medicine, University of Washington, Seattle, WA, United States; ^3^Department of Neurology, University of North Carolina at Chapel Hill, Chapel Hill, NC, United States; ^4^Department of Neurology, University of Washington, Seattle, WA, United States

**Keywords:** Alzheimer’s disease, postmortem brain, neurons, early endosome, endo-lysosomal network, confocal microscopy, high-resolution imaging, Imaris

## Abstract

Abnormal endo-lysosomal morphology is an early cytopathological feature of Alzheimer’s disease (AD) and genome-wide association studies (GWAS) have implicated genes involved in the endo-lysosomal network (ELN) as conferring increased risk for developing sporadic, late-onset AD (LOAD). Characterization of ELN pathology and the underlying pathophysiology is a promising area of translational AD research and drug development. However, rigorous study of ELN vesicles in AD and aged control brains poses a unique constellation of methodological challenges due in part to the small size of these structures and subsequent requirements for high-resolution imaging. Here we provide a detailed protocol for high-resolution 3D morphological quantification of neuronal endosomes in postmortem AD brain tissue, using immunofluorescent staining, confocal imaging with image deconvolution, and Imaris software analysis pipelines. To demonstrate these methods, we present neuronal endosome morphology data from 23 sporadic LOAD donors and one aged non-AD control donor. The techniques described here were developed across a range of AD neuropathology to best optimize these methods for future studies with large cohorts. Application of these methods in research cohorts will help advance understanding of ELN dysfunction and cytopathology in sporadic AD.

## 1 Introduction

Alzheimer’s disease (AD) is a devastating progressive neurodegenerative disorder characterized by a decline in memory and learning with variable impairment across other cognitive domains (Baker, 2016). Sporadic late-onset AD (LOAD) affects 6.7 million individuals in the US and is projected to increase in prevalence in the coming decades (Revi, 2020; Dhana et al., 2023). The social and financial burdens on families and the health care community are growing while treatments to improve symptoms or halt and reverse the disease are limited (Revi, 2020; Wegiel et al., 2021). AD neuropathologic change (ADNC) necessarily includes extracellular amyloid-beta (Aβ) plaques and intraneuronal neurofibrillary tangles (NFTs) composed of hyper-phosphorylated tau (pTau) (Montine et al., 2012). LOAD clinicopathological studies show that neuronal dysfunction, cell death, and Aβ and pTau proteinopathies occur a decade or more prior to onset of clinical signs and symptoms (Masters et al., 2015; Dubois et al., 2016). The molecular pathways involved in LOAD pathogenesis and early drivers of disease are complex and a crucial research focus for the development of effective treatments (Masters et al., 2015; Wegiel et al., 2021).

The endo-lysosomal network (ELN) is implicated in AD pathogenesis and neuronal dysfunction (Cataldo et al., 2000; Malik et al., 2019; Szabo et al., 2022). The ELN is a complex cellular trafficking system, involved in internalization of extracellular factors and trafficking of molecular cargo throughout the cell, including recycling to the cellular membrane and targeting cargo for lysosomal degradation (Malik et al., 2019). The functions of the ELN in neural cells have been extensively reviewed (Kulkarni and Maday, 2018; Szabo et al., 2022).

Abnormal endo-lysosomal morphology, for example vesicle enlargement, is a characteristic early cytopathological change in AD associated with endosomal “traffic jams” that cause neuronal dysfunction (Cataldo et al., 1997, 2000; Botte et al., 2020; Knupp et al., 2020; Mishra et al., 2022). ELN abnormalities have also been described in peripheral blood mononuclear cells, dermal fibroblasts and human induced pluripotent stem cell (hiPSC)-derived neurons sampled from LOAD patients (Israel et al., 2012; Corlier et al., 2015; Xicota et al., 2023).

**Figure 1:**
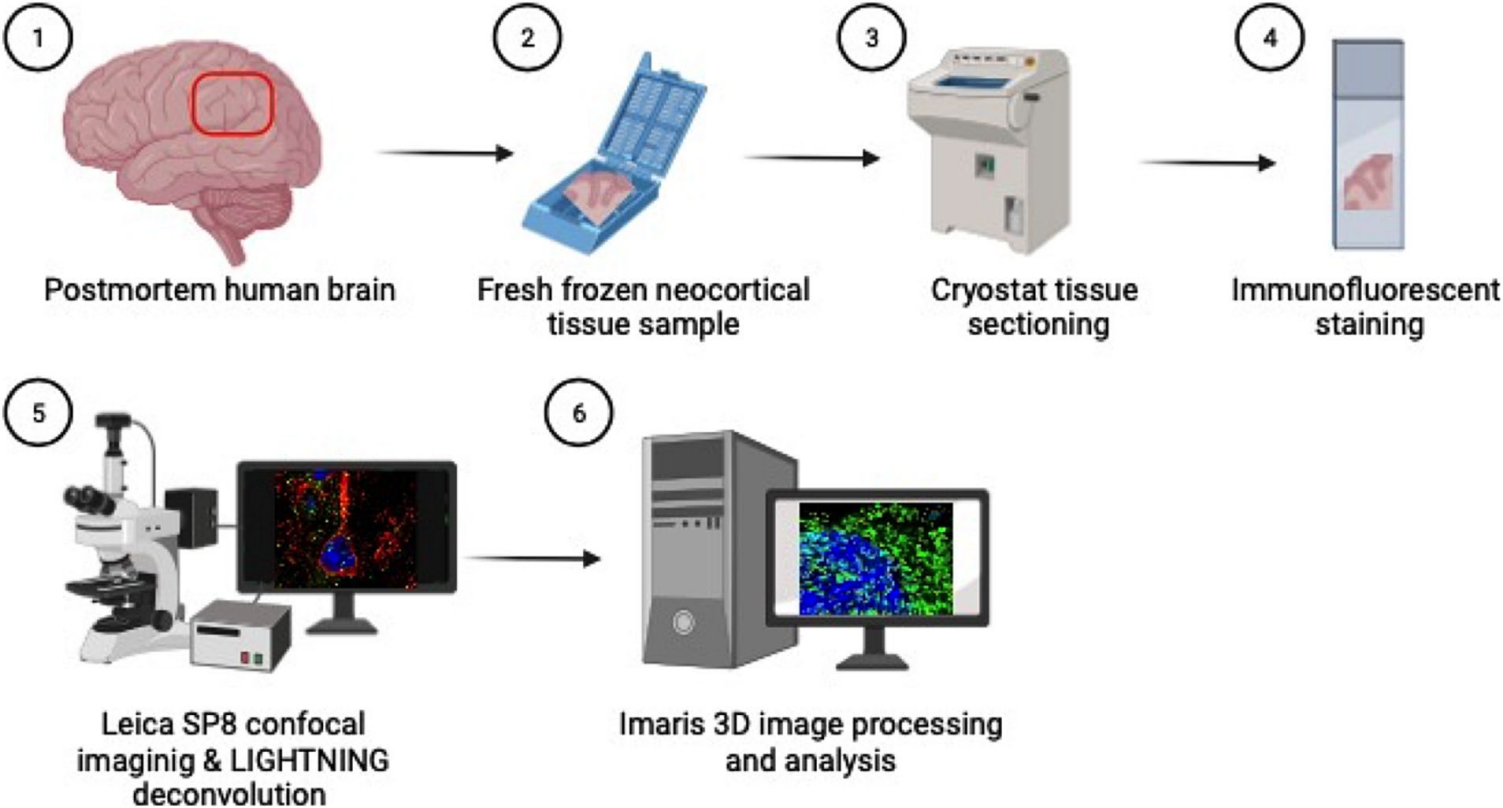
Methods overview: (1) Inferior parietal lobule brain tissue is sampled at autopsy in coronal sections (2) and fresh tissue is flash frozen in cassettes. (3) Fresh frozen tissue samples are then sectioned on a cryostat at 5 μm thick, (4) mounted on glass slides and immunofluorescently stained for cortical and endosomal markers. (5) Neuron images are acquired using the Leica SP8 confocal microscope with LAS X LIGHTNING deconvolution and (6) Imaris software is used for 3D image reconstruction and quantitative analysis of neuronal endosomes. Created with BioRender.com.

ELN dysfunction is present at early preclinical stages of LOAD and in young individuals with trisomy 21 (Down’s Syndrome) prior to dementia onset due to the duplication of *APP* on chromosome 21 (Cataldo et al., 2000; Botte et al., 2020). Enlarged early endosomes are observed in cortical neurons from postmortem prefrontal cortex at the preclinical stages of sporadic AD, where AD pathology is only limited to the entorhinal cortex and no cognitive impairment is present (Cataldo et al., 2000). Rigorous quantitation of ELN morphology is currently under-utilized in human postmortem LOAD brain tissue studies. Studies examining ELN cytopathology in AD and other neurodegenerative diseases using postmortem human brain tissue have primarily been published using small cohorts, lower-resolution imaging, or are largely descriptive (Cataldo et al., 1990, 1996, 1997, 2000; Piras et al., 2016; Chai et al., 2019; Wang et al., 2020; Sharoar et al., 2021). To address this gap in knowledge we have developed protocols to quantitatively assess 3D morphology of ELN compartments in human brain tissue at high-resolution using advancements in immunofluorescent staining, confocal imaging, and analysis software (Figure 1). The scalability of these methods to larger autopsy-based AD study cohorts is of critical importance is, given the challenges involved in high-resolution immunofluorescent imaging of postmortem brain tissue from aged brains, including tissue quality, presence of lipofuscin autofluorescence, and immunofluorescent staining. As such, we applied the methods presented here in a larger case series representing a range of AD neuropathology. We also highlight the importance of larger cohorts for adequate statistical power when studying LOAD. Finally, we show that advancements in microscopy and application of image analysis software can provide feasible methods to successfully scale to large postmortem LOAD cohorts.

## 2 Materials and equipment

### 2.1 Reagents

- Tissue Tek O.C.T. compound (Sakura Finetek USA # 4583)
- 16% formaldehyde (PFA) solution, methanol-free (Thermo Scientific # 28908)
- DPBS 1X with calcium, magnesium (Gibco # 14040-117)
- Triton X-100 (Fisher Bioreagents # BP151-100)
- Tween20 (Sigma-Aldrich # P1379)
- Normal Goat Serum (NGS) (Jackson ImmunoResearch Laboratories # 005-000-121)
- Bovine Serum Albumin (BSA) (Sigma-Aldrich # A3294)
- Mouse anti-EEA1 antibody (BD Biosciences # 610456)
- Chicken anti-MAP2 antibody (Abcam # ab92434)
- Mouse anti-LR11 (SORLA) antibody (BD Transduction Laboratories # 611861)
- Goat anti-Chicken IgY (H+L), Secondary Antibody Alexa Fluor™ 594 (Invitrogen # A11042)
- Goat anti-Mouse IgG (H+L) Secondary Antibody Alexa Fluor™ 488 (Invitrogen # A11029)
- TrueBlack Lipofuscin Autofluorescence Quencher (Biotium # 23007)
- 70% Ethanol solution (Sigma-Aldrich # 65350-M)
- ProLong Gold Antifade Mountant with DNA Stain DAPI (Invitrogen # P36931)

### 2.2 Materials/equipment

- Cryostat, model CM1850 (Leica Biosystems)
- Microscope 5-Slide Mailers, for antibody incubations (PulmoLab #240-3074-03)
- Microscope slides, positively charged, 25x75x1 mm (Globe Scientific # 1358W)
- No. 1.5 Coverglass, 24×50 mm, (VWR # 48393-241)
- ImmEdge Hydrophobic Barrier Pen (Vector Laboratories, # H-4000)
- Leica TCS SP8 confocal laser microscope with 63x/1.40 oil lens (Leica Microsystems)

### 2.3 Solution formulations

- 4% PFA: 1:4 dilution of 16% PFA stock solution in 1X DPBS.
- Blocking solution: 5% NGS + 2% BSA + 0.1% Triton X-100, in 1X DPBS.
- Primary antibody solutions:
  ○ Anti-EEA1 primary antibody at 1:500 dilution + anti-MAP2 primary antibody at 1:500, in blocking solution.
  ○ Anti-LR11 (SORLA) primary antibody at 1:1000 + anti-MAP2 primary antibody at 1:500, in blocking solution.
- Secondary antibody solution: goat anti-mouse Alexa Fluor™ 488 and goat anti-chicken 594 secondary antibodies at 1:1000 dilution +0.5% Tween20, in 1X DPBS.
- TrueBlack solution: 1:20 dilution of stock TrueBlack in 70% ethanol.

### 2.4 Software and computer

- Leica Application Suite X 3.5.5.19976 with LIGHTNING adaptive deconvolution (Leica Microsystems)
- Imaris 9.9.0 (Oxford Instruments) running on a Windows 10 system with an AMD Ryzen Threadripper 32-Core processor (3.69 GHz), 256GB of RAM, and an NVIDIA GeForce RTX 3090 24GB Graphics Card.

## 3 Methods

### 3.1 Post-mortem brain tissue collection, preservation, sampling, and evaluation

Postmortem brain tissue samples were provided by the University of Washington BioRepository and Integrated Neuropathology (UW BRaIN) laboratory which supports the UW Alzheimer’s Disease Research Center (ADRC) and the Kaiser Permanente Washington Health Research Institute Adult Changes in Thought (ACT) Study neuropathology cores. Demographic, clinical, cognitive, pharmacological, genomic, and neuropathological data were provided by the BRaIN Lab. Research donor brain collection, dissection, processing, and preservation were performed by the UW BRaIN Lab according to established protocols that meet or exceed consensus protocols (Montine et al., 2012; Latimer et al., 2023). For cases with postmortem interval (PMI) <12 h one hemisphere of the fresh brain was coronally sectioned and samples from standardized regions were dissected, placed into cassettes, flash frozen in liquid nitrogen, and stored at −80° C. Donors undergo comprehensive sampling and neuropathological workup that meet or exceeds current consensus guidelines (Montine et al., 2012; Latimer et al., 2023; Nelson et al., 2023). All relevant protocols and procedures were approved by the UW Institutional Review Board.

### 3.2 Case series selection

A total of twenty-four brain donors were selected from the UW BRaIN lab to develop the staining protocols and image analysis pipelines (Supplementary Table S1). Twenty-three cases had AD pathology present (ADNC low, intermediate or high). A representative cognitively normal non-AD (ADNC Not) control case was also included. Inferior parietal lobule cortex was chosen because it is pathologically involved by intermediate and high ADNC, correlates well with cognitive impairment, and is included in the consensus neuropathology diagnostic workup (Montine et al., 2012; Latimer et al., 2023).

### 3.3 Brain tissue preparation

For immunostaining workups, a combination of fresh frozen tissue samples from anterior temporal, inferior parietal, and occipital cortical regions from a smaller number of AD cases were used. Antibody workup experiments were also attempted in formalin-fixed paraffin-embedded (FFPE) tissue, but fresh frozen tissue studies are presented here because EEA1 early endosome staining was unsuccessful in FFPE tissue. Following antibody optimization, staining was performed on the full 24 case series in parietal cortex.

### 3.4 Brain tissue cutting and fixation

Fresh frozen neocortical brain samples 2.5 × 2.5 cm were sectioned on a cryostat at 5 μm thickness, mounted on charged glass slides, and stored at −80° C. When ready for use, tissue slides were allowed to thaw briefly, fixed with cold 4% PFA in DPBS for 45 min and washed 3x with DPBS.

### 3.5 Stepwise immunostaining protocol

#### 3.5.1 Day one

(1) Following DPBS washes in 3.4 above, incubate slides in blocking solution for 1 h at room temperature (RT) in slide mailers.
(2) Transfer slides into primary antibody solution in new slide mailers and incubate overnight at 4°C.

#### 3.5.2 Day two

(3) Wash slides 3× 5 min, in DPBS.
(4) Transfer slides into secondary antibody solution in new slide mailers and incubate for 1 h at RT in dark. Slides should be kept in the dark as much as possible hereafter to prevent immunofluorescent fading.
(5) Wash slides 3× 5 min, in DPBS.
(6) Quench autofluorescence with TrueBlack solution at 1:20 for 50 s, using pap pen (300–400 μL TrueBlack solution per slide). This step is best performed in batches of eight-ten slides at a time to prevent over-incubation.
(7) Tap off as much TrueBlack solution from slide as possible on a paper towel and wash 3× 5 min in DPBS.
(8) Tap off excess DPBS from slide on clean paper towel, use Kimwipe or cotton tipped applicator to remove pap pen border, and coverslip using Prolong gold with DAPI mountant.
(9) Allow coverslipped slides to dry flat overnight in dark at RT, then store at 4° C until ready for imaging.

### 3.6 Confocal imaging

#### 3.6.1 Leica SP8 instrument settings

Imaging was performed on a Leica TCS SP8 confocal laser microscope using a 63x/1.40 oil lens. Z-stack images were acquired in the Leica Application Suite X (LAS X 3.5.5.19976) with LIGHTNING adaptive deconvolution (Leica Microsystems) at 5x zoom magnification to best capture the spatial resolution of the small endosomal compartments. Imaging and analysis were performed blinded to all case data.

#### 3.6.2 LAS X software settings

Line sequential scanning (Sequence1: 405 nm & 552 nm, Sequence2:488 nm) were selected to prevent spectral crosstalk and maximize captured emission. Laser and gain settings were optimized using multiple representative cases to utilize the detector dynamic range while avoiding oversaturated pixels. Settings were kept constant across all cases imaged for this study. Settings were as follows: (1) DAPI on HyD detector1: 405 nm laser at 1%, gain at 483%, (2) Alexa 594 on HyD detector2: 552 nm laser at 1%, gain at 1.6%, and (3) Alexa488 on PMT: 488 nm laser power at 1.9%, gain 1,131 V.

#### 3.6.3 LIGHTNING deconvolution settings

LAS X LIGHTNING image acquisition parameters were set to the “Adaptive” strategy with “Speed/Resolution” slider bar set to maximum resolution. The mounting medium selected was “Prolong Gold” with a refractive index of 1.47. The image format was set to 1,256 × 1,256, speed 400 Hz, line average 4, zoom factor 5, and post-processed bit depth 16. The pixel size was 0.03 μm x 0.03 μm x 0.13 μm in xyz. Z-stack parameters were optimized for LIGHTNING deconvolution (by selecting “System Optimized” in Z-stack settings). Total z-size was 1.68 μm with 14 steps and a z-step size of 0.13 μm. These image capture settings achieved best practice oversampling guidelines, in which the smallest resolvable object in the image is oversampled at least two times in x and y directions (Cromey, 2010).

### 3.7 Neuron selection

For this study eight to twelve neurons were imaged from each case. Neuron number per case varied depending on tissue sample size and relative gray: white matter area. Staining two adjacent 5 μm sections per case is recommended to ensure sufficient neuron imaging numbers, if resources allow.

**Figure 2:**
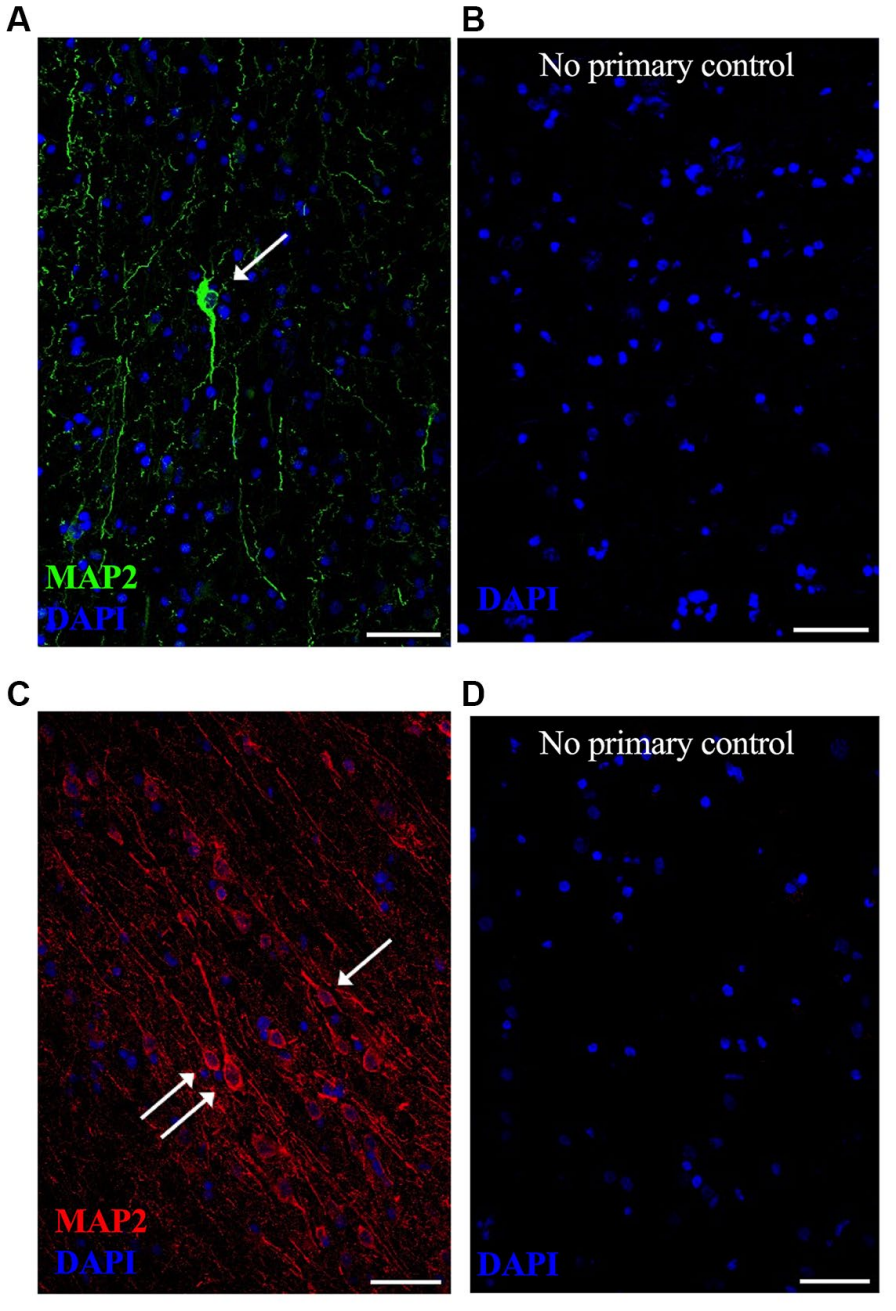
Representative lower magnification immunofluorescent images of neurons in postmortem fresh frozen cortical regions from an AD case used specifically for immunostaining methods development. MAP2 neuronal staining was equally successful in **(A)** anterior temporal cortex using Alexa488 (green) secondary antibody and **(C)** occipital cortex using Alexa594 (red) secondary antibody. Respective “no primary antibody” control conditions were run simultaneously on adjacent tissue sections, shown in **(B,D)**. Nuclei are stained with DAPI (blue). Representative image settings adjusted for illustration purposes only in figure; all settings were kept consistent between MAP2 images and respective controls [**(A,B)** pair and **(C,D)** pair]. (→) show neurons that meet imaging quality criteria. Scale bars = 50 μm.

**Figure 3:**
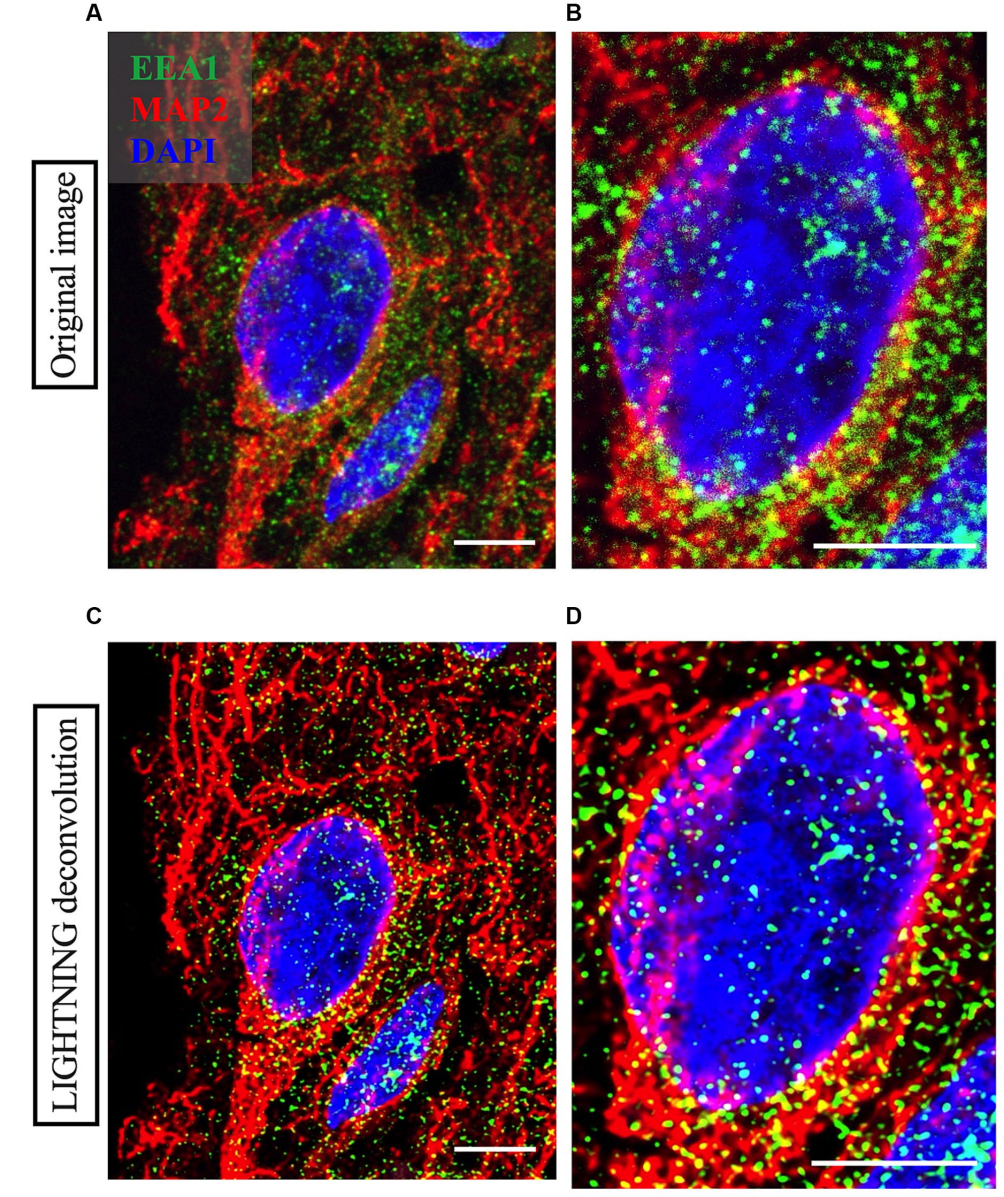
Representative high magnification images of a neuron imaged from Case 9 parietal cortex, with cortical neuron marker MAP2 in red, early endosome marker EEA1 in green and DAPI nuclear marker in blue. Comparison between the raw image **(A,B)** and the LIGHTNING deconvolved image **(C,D)**, highlights significantly improved image resolution and early endosome puncta clarity after correcting for light scatter with LIGHTNING deconvolution. Images on right **(B,D)** show further magnified view of neuron soma in **(A,C)** respectively. Representative images here are shown as max z-projections of original 14-step z-stacks to better visualize full neuronal structure in this figure but were kept as z-stacks for Imaris analysis. Representative image settings adjusted for illustration purposes only and kept consistent within figure. Scale bars = 5 μm.

Pyramidal neurons located in the inferior parietal lobule gray matter were imaged for this study. Intact pyramidal neurons were defined as having strong and uniform MAP2 staining in the conic shaped soma and proximal region of the apical dendrite, with an intact DAPI-positive nucleus present (Neurons noted by white arrows in Figures 2A,C, and neurons in Figure 3). Studies have shown that overall MAP2 staining is retained in AD brains across a range of pathological severity, although not in areas of the neuron containing NFTs (Ashford et al., 1998; Adlard and Vickers, 2002). Using these DAPI and MAP2 criteria, the neurons selected for analysis in this AD case series were unlikely to have been in advanced stages of neurodegeneration or have had significant NFT deposition, respectively, at the time of autopsy.

Tissue quality variability is commonly observed in human postmortem brain cohorts, likely due to factors such as agonal state, post-mortem interval length, and artifacts from freezing and tissue sectioning. It was therefore important in this study to demonstrate methodological reproducibility in a larger group of postmortem LOAD brains. Two of the 24 cases selected for this study were excluded due to poor tissue quality (Case 2 and Case 17 in Supplementary Table S1, noted with asterisks). Examples of z-frames of three neurons imaged from Case 17 are shown in Supplementary Figure S1. Neurons in the excluded cases lacked intact and continuous MAP2 staining (white arrows, Supplementary Figure S1), likely due to poor general tissue condition with substantial tissue cracking present (white asterisks, Supplementary Figure S1). In all cases stained in this study EEA1 and DAPI staining quality did not appear compromised but, due to underlying tissue integrity issues, MAP2+ neurons could not be identified and delineated with confidence in the two excluded cases.

We observed overall low levels of lipofuscin autofluorescence after TrueBlack quenching; With robust MAP2 and EEA1 antibody staining this background staining did not interfere with image analysis. Due to case-to-case variability it is recommended that all staining is completed before setting microscope and Imaris workflow parameters to ensure cases selected for workflow development are representative of the full cohort.

### 3.8 Imaris image analysis

The image analysis process can be summarized as a two-part process; First, the creation of neuronal regions-of-interest (ROIs) (called “surfaces” in the Imaris user interface) using the cellular marker stains, and second, identification and segmentation of EEA1(+) early endosome puncta within the neuronal ROIs. The Imaris image analysis pipeline generates a wealth of biologically relevant statistical data that can be employed to advance our understanding of endosomal cytopathology in AD.

**Figure 4:**
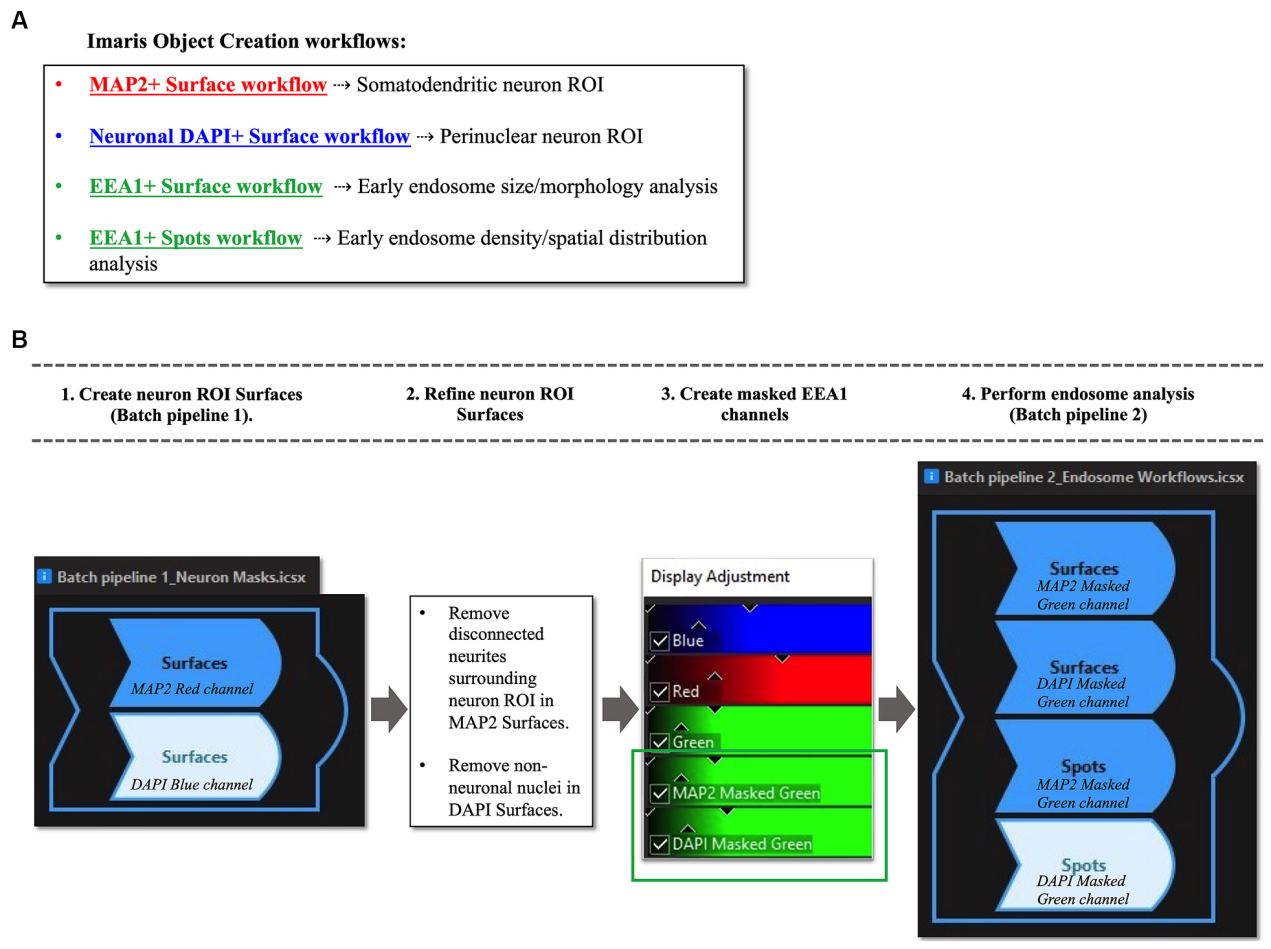
Overview of Imaris image analysis pipeline: **(A)** First, parameters are set for neuronal structure identification and segmentation via four separate “object creation” workflows using several representative z-stacks from the AD case series. Two Surface object workflows are created to define the neuronal regions-of-interest (ROIs) in which early endosomes were observed: the MAP2+ somatodendritic neuronal ROI, and neuronal DAPI+ perinuclear ROI. Both Surface object and Spots object workflows are used to gather quantitative data on EEA1+ early endosome puncta, as the Surface and Spots object workflows provide unique statistical variables related to endosome size, morphology, density, and spatial distribution within the neuronal ROIs. The workflows in **(A)** are then combined into batch pipelines, as shown in **(B)** to allow for analysis of the entire AD case cohort. First the neuronal ROIs are created using Batch Pipeline 1 (B1), after which the ROIs are refined to exclude disconnected neurites in the neuropil surrounding the neuron imaged from the MAP2+ surface and to remove non-neuronal nuclei from the DAPI+ surface (B2). The EEA1+ green channel is then masked twice over, using the two neuronal ROIs created in steps (B1-B2). Lastly, the endosome analysis batch pipeline (Batch pipeline 2) can be built, comprised of the EEA1+ surface and spots workflows created in **(A)**, with both workflows duplicated to analyze both the masked EEA1 channels (B4).

**Figure 5:**
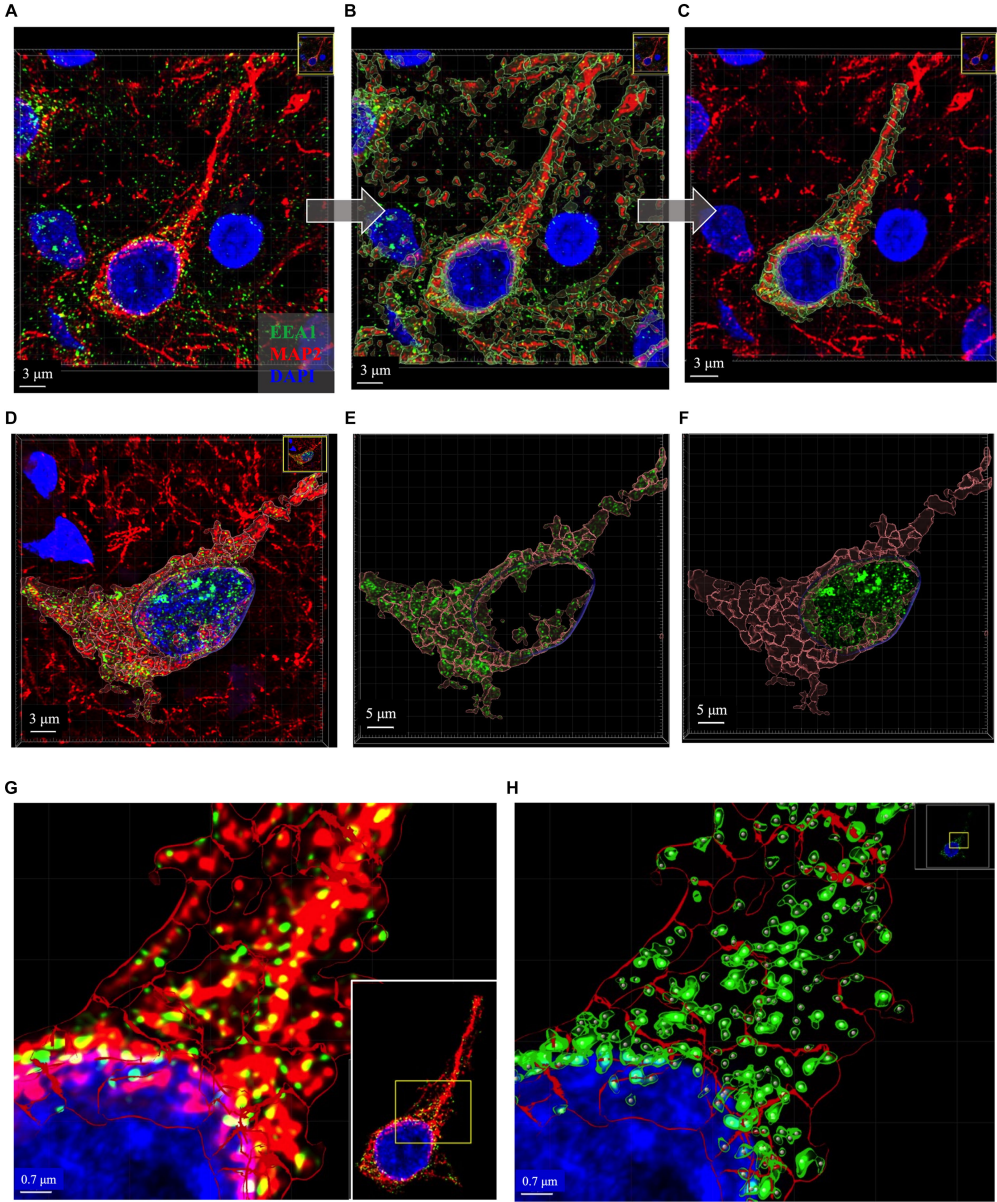
Representative images of key intermediate workflow steps. Neuron in **(A–C)** and **(G,H)** from case 21. Neuron in **(D–F)** is from Case 9. **(A–C)** Show process of creating the MAP2+ somatodendritic neuron ROI, starting with the Imaris 3D-reconstructed image **(A)**, then adding MAP+ surface object as an overlay **(B)**, removing surrounding neurites from MAP2+ surface option and using this ROI to mask the EEA1+ channel **(C)**. **(D–F)** Show representative images to illustrate generation of neuronal DAPI+ perinuclear ROI as well as the somatodendritic MAP2+ neuron ROI, and the distribution of EEA1+ puncta within each region. Varying levels of perinuclear EEA1+ staining were observed between cases, as seen in **(A)** compared to **(D)**. Masked EEA1+ fluorescent channels are shown in isolation within the MAP2+ **(E)** and DAPI+ **(F)** ROI surfaces. **(G)** Higher magnification of the punctate neuronal EEA1 staining, with magnified region corresponding to the yellow box drawn in inset image. **(H)** EEA1+ early endosome puncta are identified and segmented using the surface object workflow (green outlines around EEA1+ fluorescent puncta) and spots object workflow (gray dots centered over each EEA1+ fluorescent puncta). Images are shown in Imaris display default “3D view,” as would be seen in software during analysis process, and all image settings were kept consistent across figure. Scale bars vary in length in figure and are thus labeled accordingly in each image.

Early endosome morphology metrics from cortical neurons were analyzed with four distinct standardized workflows using Bitplane Imaris software (Oxford Instruments) (Figure 4A). Briefly, we outline how to create two neuronal ROIs, the somatodendritic neuron ROI and the perinuclear neuron ROI, within which endosomal morphology can be analyzed. The somatodendritic and perinuclear neuronal ROIs are created by generating surface objects corresponding to the MAP2(+) and DAPI(+) channels, respectively, that are then used to mask the EEA1(+) green channel (Figures 4, 5).

In the process of this study, we observed variable EEA1(+) endosomal puncta in the transitional perinuclear region between the MAP2(+) stained region and the DAPI(+) nucleus (Figures 5A–F). Upon close inspection the EEA1(+) puncta are clearly found surrounding the DAPI(+) region of the nucleus, without colocalizing with the DAPI signal, and tightly follow the invaginations and contours of the nucleus. Thus, we developed the DAPI(+) neuronal ROI to analyze these perinuclear EEA1(+) not fully captured within the MAP2(+) ROI.

The somatodendritic and perinuclear neuronal ROI surfaces described above are then used to apply a mask over the EEA1(+) green channel (Figures 5D–F) to allow for analysis of neuronal early endosomal puncta size, morphology, and distribution within the neuron via automated Imaris cell segmentation algorithms (Figure 5H). Two separate image analysis workflows offered by Imaris are used for EEA1(+) early endosome analysis: the 3D “surface” and “spots” object rendering tools (Figures 5G,H). The specific parameters used for each of the four object workflows are detailed in Supplementary Figure S2. It is recommended that a range of representative neurons sampled across the full case cohort are tested when developing these workflows before finalizing settings, due to the varying staining intensities, autofluorescence levels, and neuronal and endosomal morphologies present in human postmortem cases.

#### 3.8.1 Somatodendritic neuron ROI workflow

The “Surface” object was used to create the somatodendritic neuronal ROI by following prompts within the creation wizard to set algorithm parameters that best captured the MAP2(+) neuronal surface. The workflow was stored to add into a Batch pipeline in Section 3.8.4 below.

The initial object created includes all MAP2(+) neurites within the neuropil surrounding the primary neuron (Figure 5B). The surfaces overlayed on the surrounding distal neurites were manually deleted to generate the final ROI: the MAP2(+) neuron soma and proximal region of apical dendrite, plus any smaller branching dendrites continuous with the MAP2(+) somatic staining (Figure 5C). The MAP2(+) somatodendritic compartment surface was used to generate a second green channel showing EEA1(+) staining only within this ROI (labeled “MAP2 Masked Green” in Figure 4B, step 3).

#### 3.8.2 Perinuclear neuron ROI workflow

A Surface object workflow is also used to create the neuronal DAPI(+) ROI, similar to Section 3.8.1 above, except using the DAPI channel. Non-neuronal DAP1(+) nuclei were manually deleted after the surface creation (Figures 5D,F). This neuronal DAPI(+) surface is then used to mask on original EEA1 green channel, generating a third green EEA1 channel specific to the neuronal perinuclear ROI (labeled “DAPI Masked Green” in Figure 4B, step 3).

#### 3.8.3 Early endosome workflows

The EEA1(+) surface creation workflow followed the same process as the neuron ROI steps above in Sections 3.8.1 and 3.8.2, with particular care given to accurately segment individual EEA1 puncta. The EEA1(+) surfaces generated are shown in Figure 5H as green outlines surrounding EEA1(+) fluorescent puncta. The EEA1(+) spots workflow involves a similar stepwise creation wizard but generates dots that correspond to each EEA1(+) fluorescent puncta (Figure 5H, gray dots).

The minimum value for early endosome volume in the Imaris EEA1(+) surface object workflow was based on published early endosome diameter measurements by EM (Klumperman and Raposo, 2014). This was done to standardize our analysis methods and ensure inter-researcher reproducibility versus relying on what a researcher determines by eye to be “real staining.” To our knowledge, there are no generally agreed upon published size ranges for human early endosome by immunofluorescent confocal imaging, likely due to the wide range of tissue fixation methods as well as immunofluorescent imaging techniques. Many publications do not describe a minimum volume; it is unclear in most cases if that detail was simply not included in the methods or if no lower bound to volume was employed. By EM, human endosomes range from 100 to 500 nm in diameter (Klumperman and Raposo, 2014). To calculate the minimum volume from a diameter measurement we assumed that endosomes are spherical. Therefore, EEA1(+) fluorescent puncta with volumes <0.004 μm^3^ were excluded.

#### 3.8.4 Batch pipelines

Once the four individual object workflows outlined above have been created and saved (Figure 4A) they can be combined into Batch pipelines and applied to the full image dataset (Figure 4B). This allows for the application of the same object workflow creation parameters to a large image dataset for high-throughput analysis. In our protocol, we created two separate Batch pipelines, one to define the neuronal ROIs (“Batch Pipeline 1” in Figure 4B) and a second to identify, segment and analyze the early endosomes within these two compartments (“Batch Pipeline 2” in Figure 4B). It was necessary to divide the object creation workflows in this manner to carry out the neuronal surface ROI mask editing and creation of masked EEA1 channels (Steps #2 and #3 in Figure 4B).

In Batch Pipeline 2 (Figure 4, step 4) all EEA1(+) workflows created in previous steps are compiled into a single image analysis pipeline that can be applied to the full image data set, where each step of the pipeline corresponds to individual EEA1 object creation workflows. A statistical data report is generated from each batch pipeline.

## 4 Results

**Figure 6:**
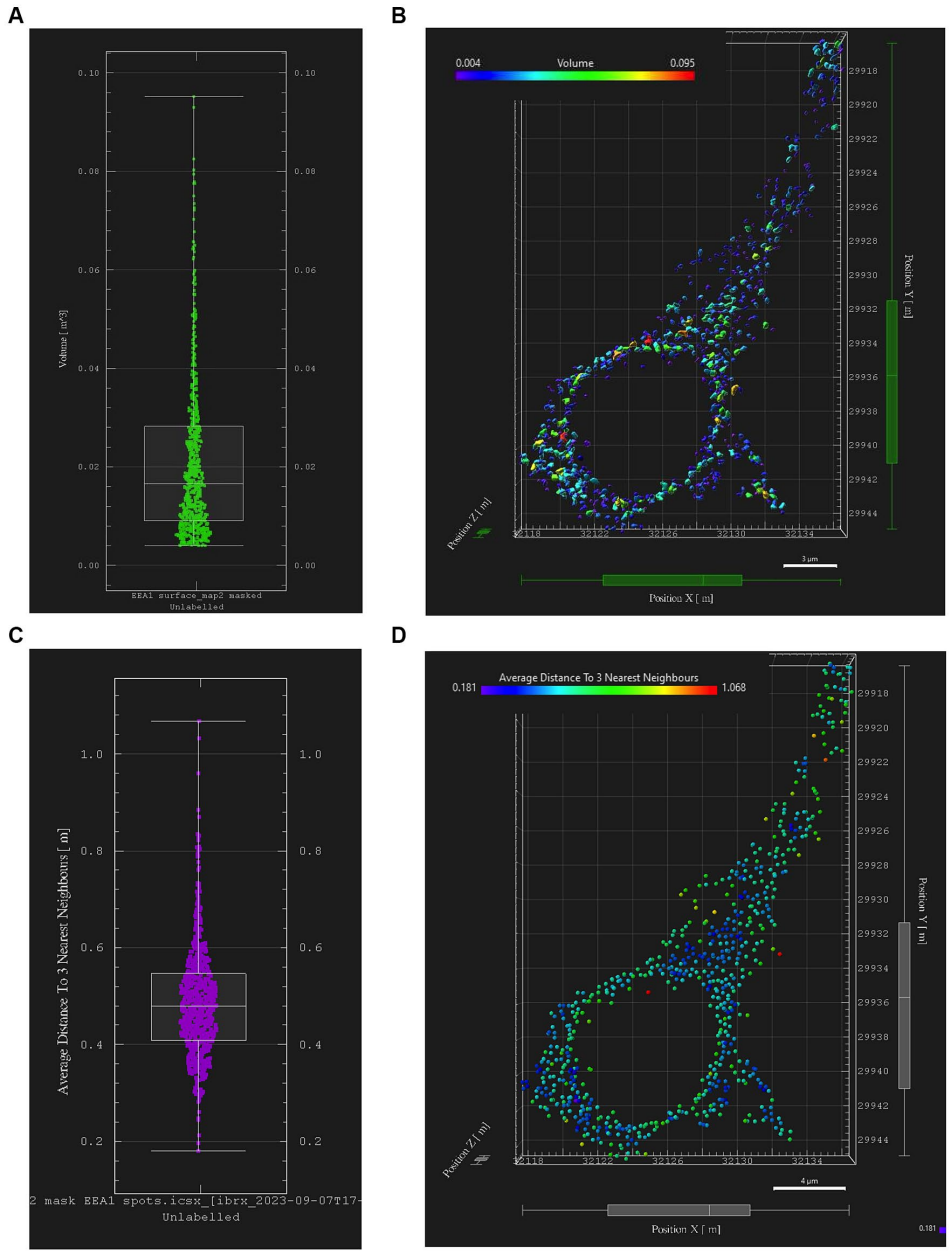
Early endosome image analysis data from the somatodendritic neuron ROI of a representative neuron (Case 21, image also used in Figure 5). Graphs and images were generated with Imaris “Vantage View,” an interactive statistical data visualization module of the software. **(A)** Box plot showing early endosome puncta volumes; white bars are range, median, Q1 and Q3. **(B)** Scatter plot showing xyz spatial distribution of early endosome surfaces with somatodendritic neuron ROI, color-coded by puncta volume. **(C)** Box plot showing “mean distance of three nearest neighboring endosomes” within ROI, a statistical output aimed at quantifying early endosome clustering “tightness” and early endosome densities; white bars are range, median, Q1 and Q3. **(D)** Scatter plot showing xyz spatial distribution of EEA1+ early endosomes color-coded by “mean distance of three nearest neighbors” value within the somatodendritic neuron ROI.

Early endosome data and statistics can be visualized in Imaris through the interactive Vantage plot software module (Figure 6). Consistent with studies of *in vitro* and *in vivo* cellular endosomal size in the literature, we observed a “flame-shaped” early endosome volume distribution, with the frequency skewed toward a greater proportion of smaller endosome volumes (Figure 6A). Viewing endosomal volumes in xyz space within the somatodendritic space (Figure 6B) has the potential to reveal insights into the relationship between endosome size, morphology, and localization within the neuron or other cell of interest.

**Figure 7:**
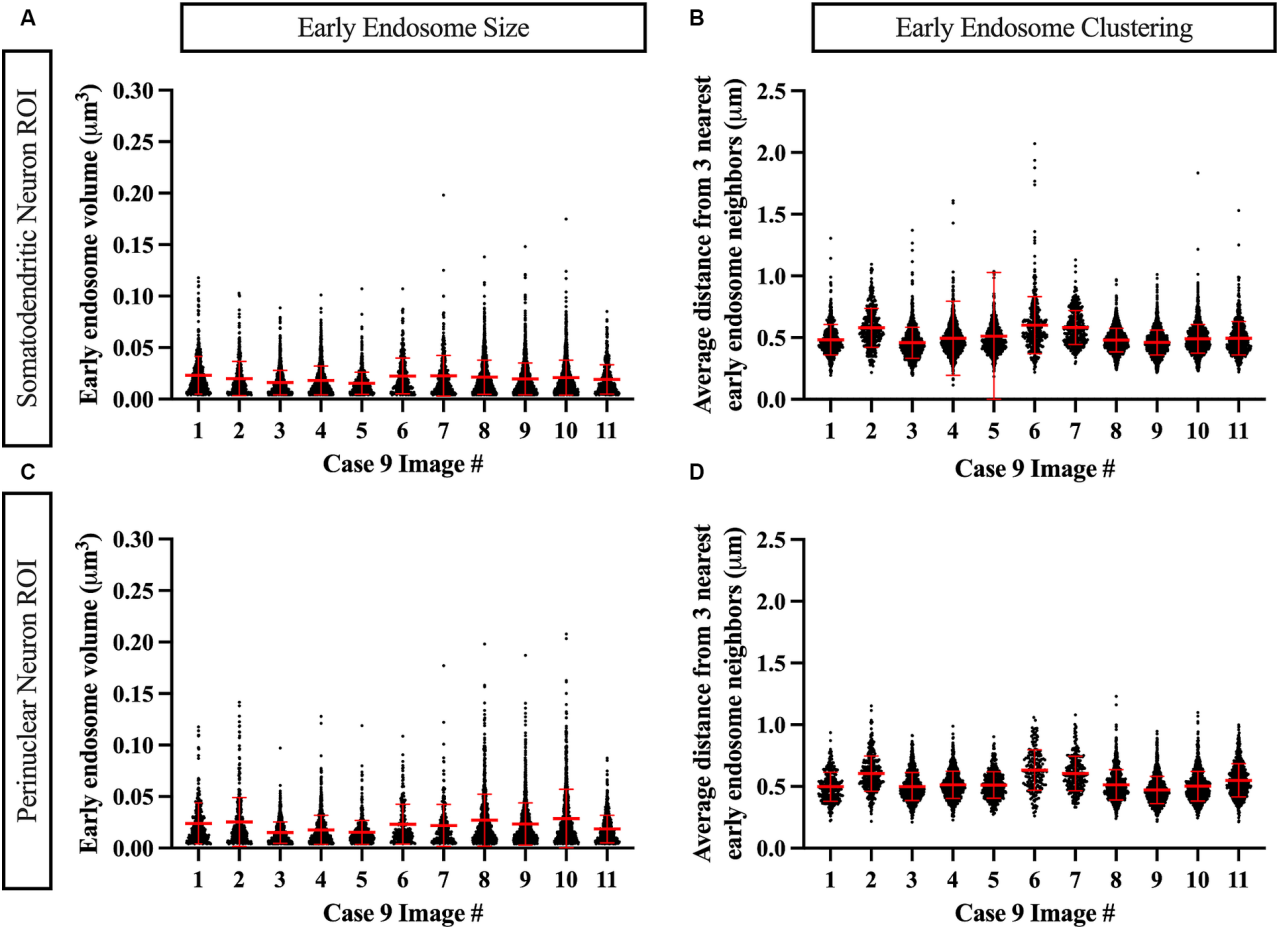
Example data output from Imaris image analysis, shown from Case 9 neurons (eleven images), in both the MAP2+ somatodendritic neuron ROI **(A,B)** and the DAPI+ perinuclear neuron ROI **(C,D)**. **(A,C)** Early endosome puncta volume measurements per image. **(B,D)** Endosome clustering measurements, via average distance from three nearest early endosome neighbors, per image. Red bars show mean and standard deviation.

Similarly, data generated from the EEA1 spots object creation can be visualized using the Vantage module. The representative data variable from the spots object workflow shown in Figure 6C is the mean distance measurement of the three early endosome puncta nearest to each punctum identified by the workflow. In addition, these localized clustering statistics can be mapped within the xyz space of the neuronal ROI (Figure 6D) to gather information regarding spatial differences in endosome positioning and density, which can provide more granular and specific information regarding where in the neuron endosomal physiology may be affected most severely, as reflected by changes in the endosomal cytopathology. Early endosome volume and clustering data in both somatodendritic and perinuclear neuronal ROIs for each of the eleven images from Case 9 are shown in Figure 7. A representative non-AD cognitively intact control case (Case 24, Supplementary Table S1) was also included in these analyses. Early endosome volume and clustering data from the eleven images captured from this control case, within both the somatodendritic and perinuclear neuronal compartments, is presented in Supplementary Figure S3. No obvious differences in staining quality, imaging or performance of the analysis workflow were observed when analyzing the control case compared the AD case series.

**Figure 8:**
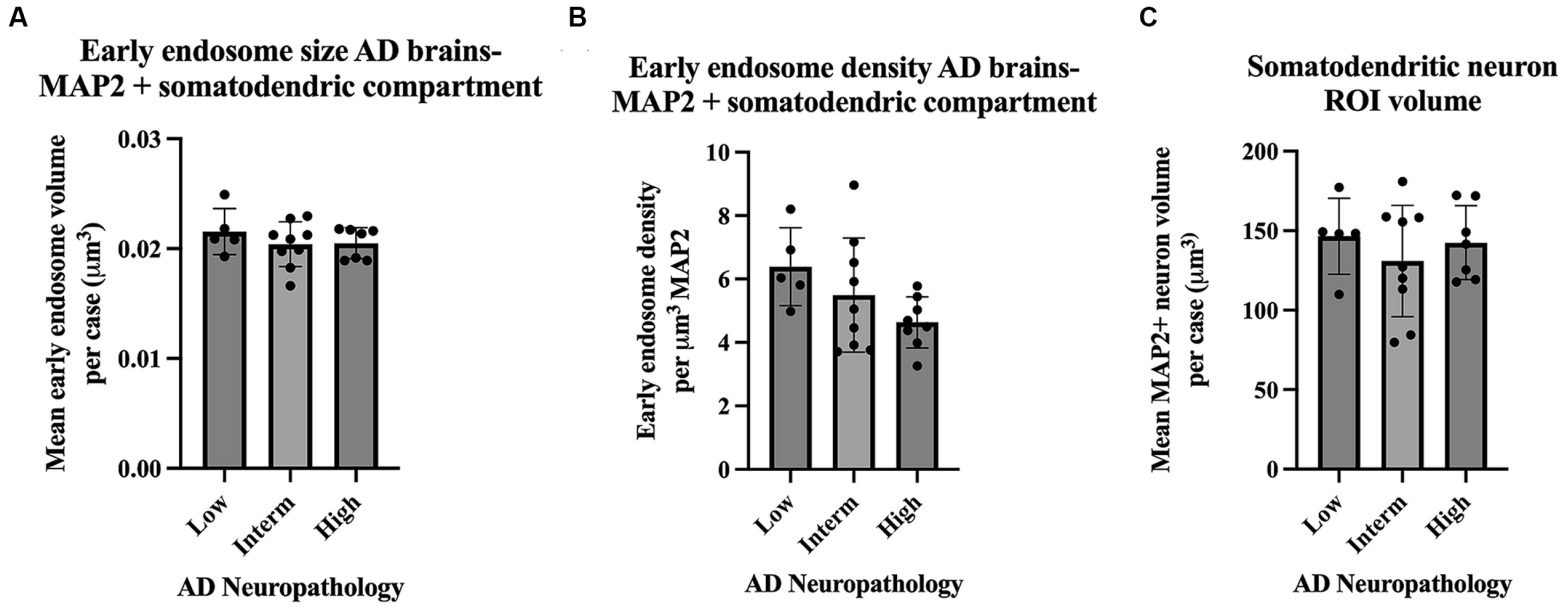
Example data output from Imaris early endosome analysis within the somatodendritic neuron ROI, shown from the full AD case series (21 cases). Cases are broken down by AD neuropathologic change (ADNC) low, intermediate (interm) and high for illustrative purposes only. Graphs show several data variables analyzed: **(A)** Early endosome volumes, **(B)** early endosome density within ROI, and (C) MAP2+ neuron volumes. For all graphs in this figure, each data point represents the mean value from all neurons imaged per case. Seven to twelve neurons were imaged per case. Bar graphs show mean and standard deviation.

Assays of large cohorts are necessary to accurately capture the range and complexity of neuronal cytopathologies observed in LOAD brains. We show data analysis collected from 21 AD cases to demonstrate as proof of concept that it is feasible to apply these methods to a larger human tissue case series (Figure 8). The AD case series data in Figure 8 is separated by ADNC low, intermediate (interm), and high for descriptive purposes only, to show these representative data outputs. Several metrics quantified from the early endosomes within the MAP2(+) somatodendritic compartment were selected as example outputs of these techniques, including mean early endosome volume per case (Figure 8A), mean early endosome density per case (Figure 8B), as well as mean MAP2(+) neuron ROI volume (Figure 8C). Future studies including non-diseased controls will be needed to make disease-relevant conclusions from these data.

**Figure 9:**
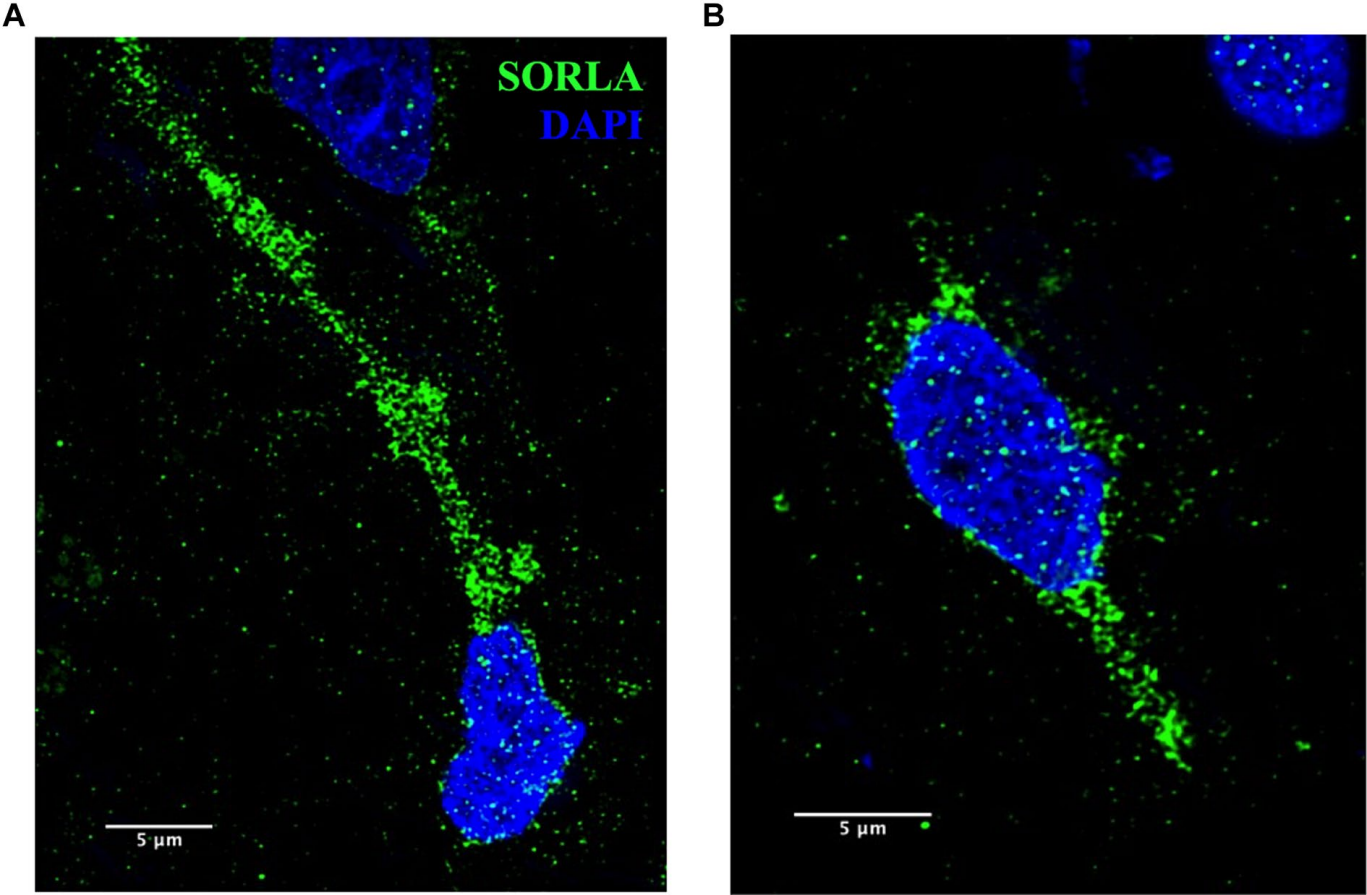
**(A,B)** Representative SORLA staining (green) in occipital cortex from AD brain. Nuclei are stained with DAPI (blue). Representative image settings adjusted for illustration purposes only and kept consistent within figure. Scale bars = 5 μm.

These methods are also applicable to other ELN components and will be useful to analyze disease-specific endosomal proteins implicated in AD, such as SORLA, shown in Figure 9. SORLA, encoded by the gene *SORL1*, is highly implicated in AD risk and is a specific endosomal sorting receptor that traffics cellular cargo, such as the amyloid precursor protein, via the retrograde and recycling endosomal pathways (Knupp et al., 2020; Andersen et al., 2022; Mishra et al., 2022; Szabo et al., 2022; Jensen et al., 2023).

## 5 Discussion

Here we show high-resolution 3D visualization of endosomes in multiple regional compartments of the neuron in a large series of postmortem sporadic LOAD brains. Using Imaris analysis software pipelines we can perform precise quantification and integrated statistical analysis of disease-relevant endosome morphology variables. Importantly, we successfully analyzed 22 out of the 24 postmortem brains in this series, showing that these methods are sufficiently robust to accommodate tissue quality variability and other technical obstacles commonly encountered in studies using postmortem brain tissue.

ELN morphological studies using human postmortem brain tissue in AD and related diseases cohorts have primarily been conducted using immunohistochemistry and microscopy techniques with lower resolution images (Cataldo et al., 1996, 1997, 2000; Piras et al., 2016; Chai et al., 2019). Several recent human *in vitro* studies using electron microscopy (EM) or super-resolution structured illumination microscopy (SR-SIM) to examine ELN morphology *in vitro* have shown that increased endosome vesicle clustering and density, rather than enlargement of individual vesicles, are associated with AD and Down’s syndrome (Botte et al., 2020; Xicota et al., 2023). Additionally, a recent publication focusing on quantification of neuronal vesicular compartments in mouse brains compared ImageJ and Imaris software capabilities and found that Imaris more accurately segmented individual vesicles, required less manual editing, and was more time-efficient (Gautier and Ginsberg, 2021). These studies highlight the importance of high-quality imaging and techniques when studying ELN compartments to accurately identify the specific ELN abnormalities occurring in AD. High-resolution imaging techniques such as EM or SR-SIM may not be widely available to researchers. Developing image analysis methods on confocal microscopes with the addition of deconvolution, as we show here, allows these methods to be more widely adopted. Imaris software analysis capabilities allow comprehensive data analysis beyond simply measuring vesicle size including variables related to regional and spatial distribution. We provide detailed transparent and reproducible image processing and analysis protocols, methods often glossed over in publications, that will allow researchers to easily access the methodological tools required to study the ELN at high resolution in human postmortem brain tissue.

Characterization of ELN pathology in large human AD studies is critical to advance our understanding of AD disease processes. The molecular underpinnings of LOAD are complex and the earliest pathogenesis steps, which occur years prior to onset of clinical symptoms, are difficult to study. Studies using postmortem human brain tissue have several inherent limitations, as the data collected represents a single cross-sectional time point and does not allow for disease modeling, intervention studies or “real-time” investigation of endo-lysosomal phenotypes with disease progression. Future studies on human iPSC-derived central nervous system cell types provide encouraging avenues to expand AD research in areas of study not well addressed with postmortem AD brain tissue. Nevertheless, the application of these methods will aid in our understanding of ELN cytopathology as it relates to development of hallmark AD pathologies and other measures of clinical and neuropathological disease progression. These protocols also provide a framework to leverage additional advances in imaging and image analysis, including superresolution nanometer-scale microscopy techniques and machine-learning approaches, to resolve and identify endosomes and other small-scale structures in large data sets in a high-throughput manner. The methods described here can also be applied to the analysis of other ELN proteins and central nervous system cell types known to be involved in AD. Detailed, reproducible, and transparent image analysis protocols will allow ELN cytopathologies to be studied and compared in a consistent manner across multiple brain banks and large longitudinal neurodegenerative disease cohorts, greatly advancing our understanding of this key early AD cytopathological process.

## Data availability statement

The raw data supporting the conclusions of this article will be made available by the authors, without undue reservation.

## Ethics statement

Ethical approval was not required for the studies involving humans because the study uses de-identified postmortem tissue, which is not considered human subjects research. The studies were conducted in accordance with the local legislation and institutional requirements. The human samples used in this study were acquired from The UW Neuropathology Core, whose protocols and procedures are approved by the UW Institutional Review Board. Written informed consent to participate in this study was not required from the participants or the participants’ legal guardians/next of kin in accordance with the national legislation and the institutional requirements.

## Author contributions

SR: Conceptualization, Data curation, Formal analysis, Investigation, Methodology, Writing – original draft, Writing – review & editing. CW: Formal analysis, Writing – review & editing. DH: Conceptualization, Methodology, Writing – review & editing. SM: Conceptualization, Writing – review & editing. AK: Methodology, Writing – review & editing. CK: Conceptualization, Methodology, Resources, Writing – review & editing. GG: Conceptualization, Methodology, Funding acquisition, Writing – review & editing. SJ: Conceptualization, Funding acquisition, Writing – review & editing. JY: Conceptualization, Funding acquisition, Resources, Supervision, Writing – original draft, Writing – review & editing.

## Supplementary material

SUPPLEMENTARY Table S1 Demographic, clinical and neuropathologic data for full case series. Cases with (*) were removed from analysis after immunostaining due to poor tissue quality. LATE Stage was not assessed (NA) for Case 22.

SUPPLEMENTARY FIGURE S1 Representative z-frames **(A–C)** shown three different z-stacks imaged from Case 17. Case 17 was removed from image analysis due to poor tissue quality and MAP2 staining. (*) show regions in images where tissue has broken up and compromised the integrity of the neurons. (→) show poor and incomplete MAP2 staining, which prevented accurate ROI creation and EEA1 early endosome channel masking. Representative image settings adjusted for illustration purposes only and kept consistent within figure. Scale bars = 3 μm.

SUPPLEMENTARY FIGURE S2 **(A–D)** Imaris object creation parameters optimized for early endosome analysis in LOAD case series.

SUPPLEMENTARY FIGURE S3 Example data output from Imaris image analysis, shown from non-AD cognitively normal control Case 24 neurons (eleven images), in both the MAP2+ somatodendritic neuron ROI **(A,B)** and the DAPI+ perinuclear neuron ROI **(C,D)**. **(A,C)** Early endosome puncta volume measurements per image. **(B,D)** Endosome clustering measurements, via average distance from three nearest early endosome neighbors, per image. Red bars show mean and standard deviation.
