# Supplementary material for: Advancements in high-resolution 3D microscopy analysis of endosomal morphology in postmortem Alzheimer’s disease brains
Source: Front Neurosci. 2024 Jan 16;17:1321680. doi: 10.3389/fnins.2023.1321680 (PMC10824887; doi:10.3389/fnins.2023.1321680)
Supplement: SUPPLEMENTARY Table S1 — Demographic, clinical and neuropathologic data for full case series. Cases with (*) were removed from analysis after immunostaining due to poor tissue quality. LATE Stage was not assessed (NA) for Case 22. [file Data_Sheet_1.PDF]

Supplementary Table. Case series demographic, clinical and neuropathological data.

Case Number	Sex	APOE	Age (yrs)	Cognition	PMI (hours)	ADNC	Thal Phase	BRAAK Stage	CERAD Score	LATE Stage
Case 1	Male	3/3	90+	Normal	5.0	High	4	V	Moderate	0
*Case 2	Female	3/3	90+	Dementia	4.8	Intermediate	4	V	Sparse	3
Case 3	Female	3/3	90+	Normal	5.1	High	4	V	Frequent	0
Case 4	Male	3/3	87	Normal	3.3	High	4	V	Moderate	2
Case 5	Male	3/4	89	Normal	6.8	Intermediate	3	V	Sparse	0
Case 6	Female	3/4	74	Dementia	4.4	High	5	VI	Frequent	0
Case 7	Female	3/3	90+	Dementia	5.1	Intermediate	3	IV	Sparse	2
Case 8	Female	3/4	90+	Dementia	4.3	High	5	VI	Moderate	2
Case 9	Female	3/4	90+	Dementia	4.0	Intermediate	3	III	Moderate	0
Case 10	Female	3/3	90+	Normal	3.0	Low	2	IV	None	0
Case 11	Female	3/3	79	Normal	6.0	Low	4	II	Sparse	0
Case 12	Female	3/3	90+	Dementia	5.9	Intermediate	4	IV	Moderate	2
Case 13	Male	3/4	88	Normal	5.3	High	5	V	Moderate	2
Case 14	Female	3/3	90+	Normal	7.4	Low	1	IV	None	0
Case 15	Female	3/3	90+	Normal	3.7	Intermediate	3	V	Moderate	0
Case 16	Female	3/3	90+	Dementia	6.3	High	4	V	Moderate	2
*Case 17	Female	3/4	77	Dementia	3.3	High	5	VI	Frequent	0
Case 18	Female	3/3	86	Normal	8.1	Intermediate	4	IV	Frequent	0
Case 19	Male	3/4	88	Normal	2.5	Intermediate	5	IV	Moderate	1
Case 20	Female	3/3	90+	Dementia	5.7	Intermediate	4	IV	Moderate	1
Case 21	Male	3/3	90+	Normal	5.0	Low	2	V	Sparse	2
Case 22	Female	3/3	83	Normal	5.9	Low	1	0	None	NA
Case 23	Female	3/3	89	Normal	5.6	Intermediate	3	IV	Sparse	0
Case 24	Female	2/3	97	Normal	4.3	Not	0	IV	None	0
