# Supplementary figures and images for: Advancements in high-resolution 3D microscopy analysis of endosomal morphology in postmortem Alzheimer’s disease brains
Source: Front Neurosci. 2024 Jan 16;17:1321680. doi: 10.3389/fnins.2023.1321680 (PMC10824887; doi:10.3389/fnins.2023.1321680)

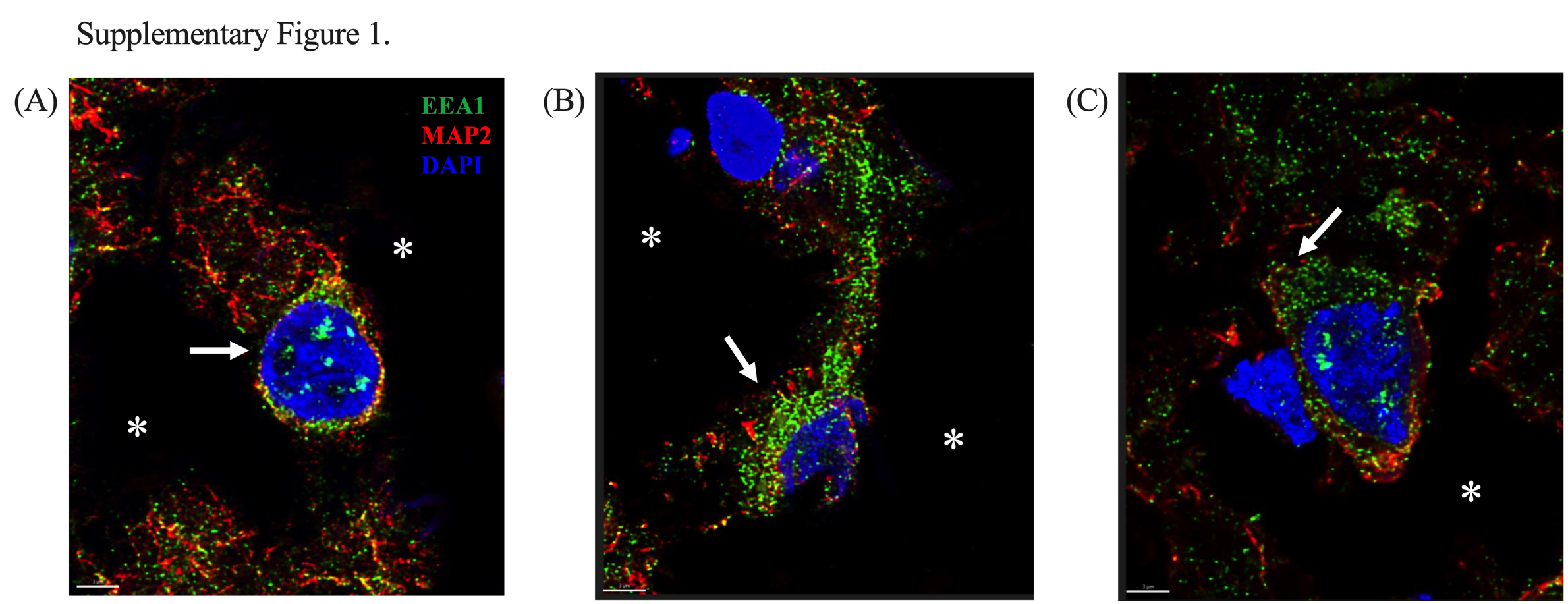

Supplement: SUPPLEMENTARY FIGURE S1 — Representative z-frames (A–C) shown three different z-stacks imaged from Case 17. Case 17 was removed from image analysis due to poor tissue quality and MAP2 staining. (*) show regions in images where tissue has broken up and compromised the integrity of the neurons. (→) show poor and incomplete MAP2 staining, which prevented accurate ROI creation and EEA1 early endosome channel masking. Representative image settings adjusted for illustration purposes only and kept consistent within figure. Scale bars = 3 μm. [file Image_1.TIFF]

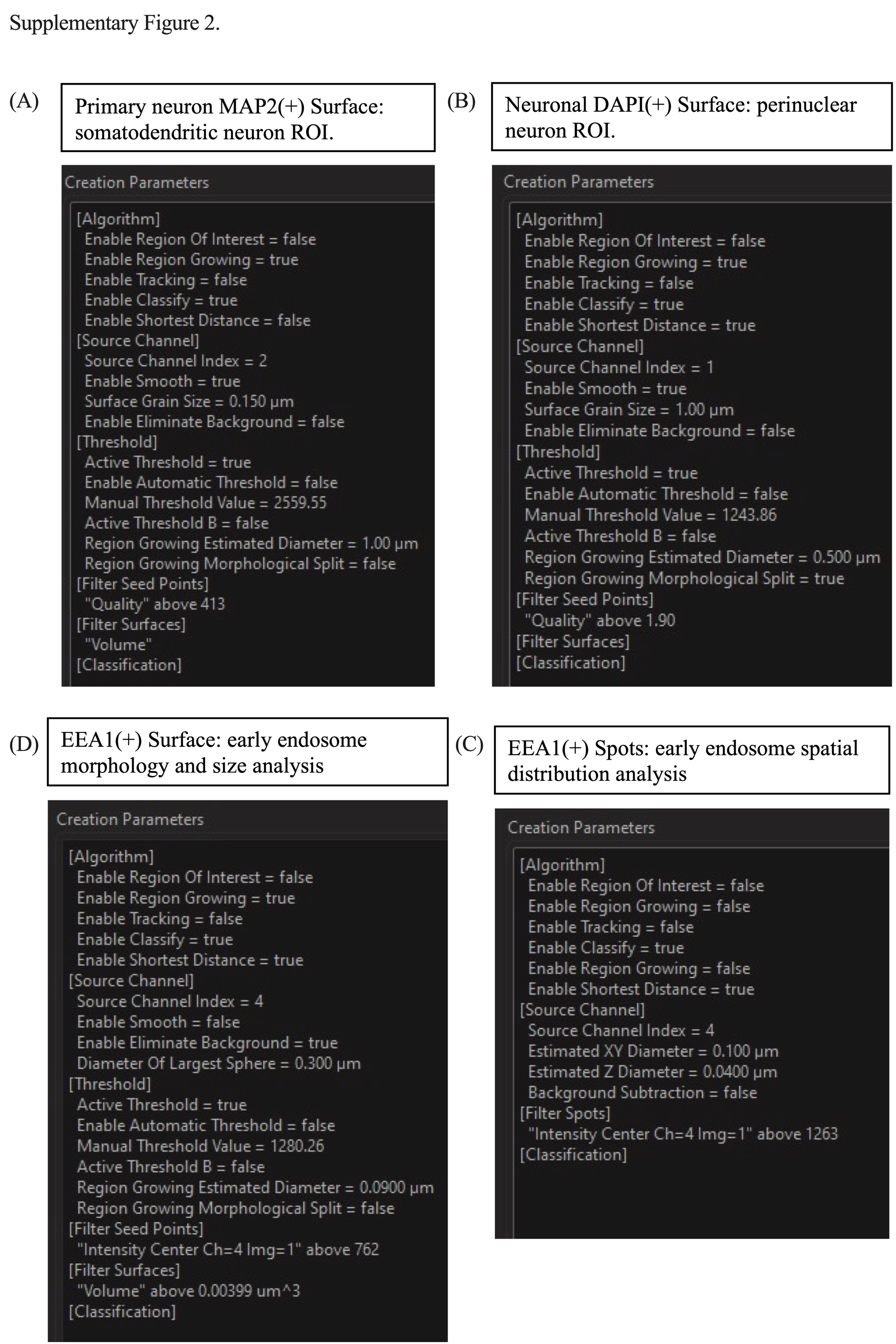

Supplement: SUPPLEMENTARY FIGURE S2 — (A–D) Imaris object creation parameters optimized for early endosome analysis in LOAD case series. [file Image_2.TIFF]

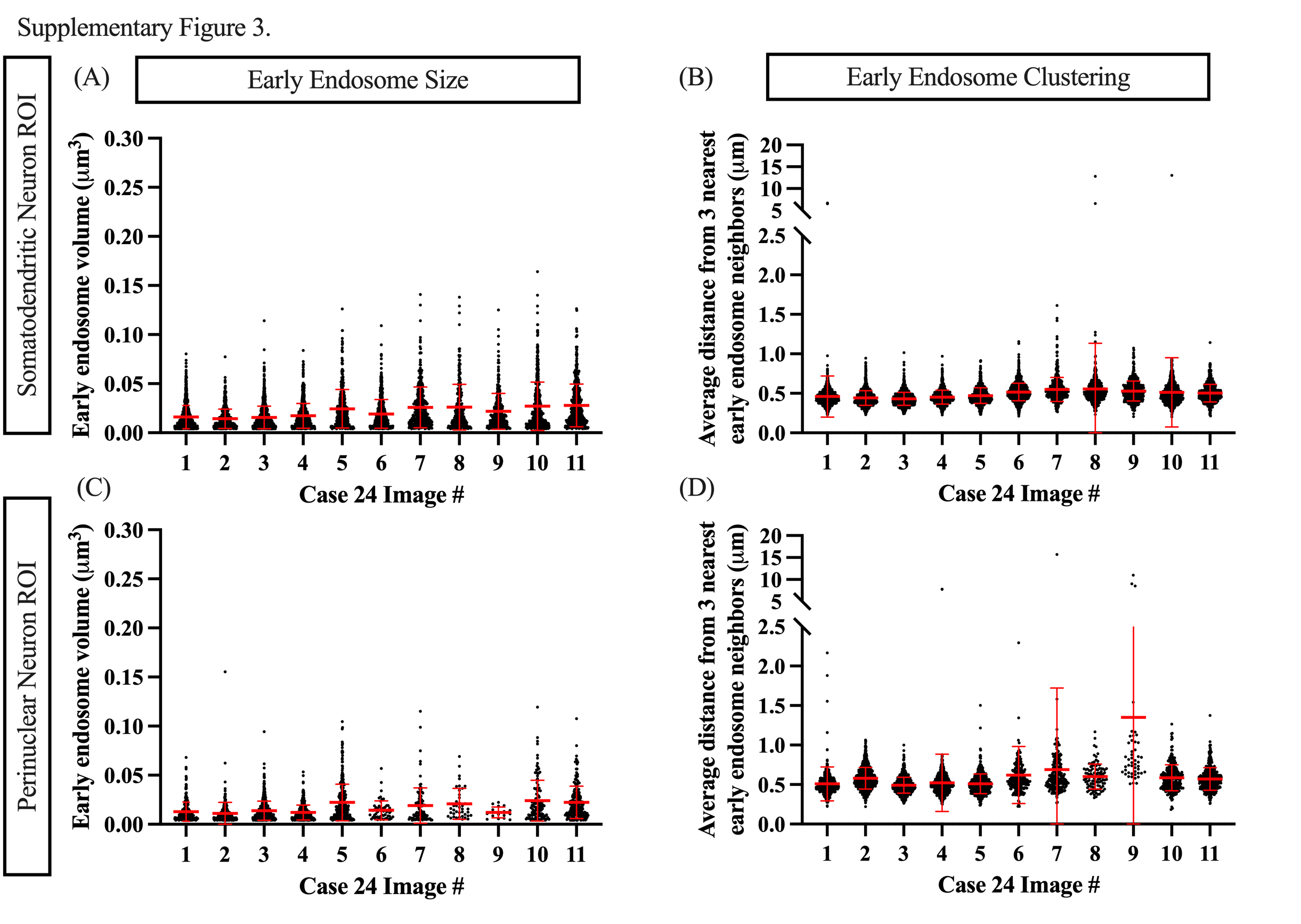

Supplement: SUPPLEMENTARY FIGURE S3 — Example data output from Imaris image analysis, shown from non-AD cognitively normal control Case 24 neurons (eleven images), in both the MAP2+ somatodendritic neuron ROI (A,B) and the DAPI+ perinuclear neuron ROI (C,D). (A,C) Early endosome puncta volume measurements per image. (B,D) Endosome clustering measurements, via average distance from three nearest early endosome neighbors, per image. Red bars show mean and standard deviation. [file Image_3.TIFF]
